# A Ring for a Ring Finger: A Case Report on Finger Prosthesis

**DOI:** 10.7759/cureus.53195

**Published:** 2024-01-29

**Authors:** Sharayu Nimonkar, Vikram Belkhode, Pranali Nimonkar, Shreyas Gotoorkar

**Affiliations:** 1 Prosthodontics, Sharad Pawar Dental College and Hospital, Datta Meghe Institute of Research and Higher Education, Wardha, IND; 2 Trauma Care Centre, Government Medical College and Hospital, Nagpur, Nagpur, IND; 3 Prosthodontics, Saraswati Dhanwantari Dental College, Parbhani, IND

**Keywords:** copper ring, amputation, silicone prosthesis, finger prosthesis, esthetics

## Abstract

The primary use of the hand is to grasp, hold, and manipulate objects. Perhaps the most obvious kind of non-verbal communication is the hand gesture. The most common types of partial hand loss are finger and partial finger amputations. Traumatic injuries, congenital absences, or abnormalities are frequent causes, and they create significant treatment issues. In addition to the acute loss of grab power, the disappearance of fingers may result in significant psychological harm. People who want their fingers replaced frequently have high expectations for how a prosthesis will look. This clinical report illustrates an easy way to keep an acrylic finger prosthetic in place.

## Introduction

Patients who suffer an injury or lose some of their structures after surgery require restoration for both aesthetics and functionality. Surgery is the main form of treatment for many conditions, but it cannot correct all of them. The restoration of some tissue abnormalities or loss is possible with prosthetics. A process of artificial replacement or reconstruction for any human body part, including fingers, eyes, ears, etc., is called prosthetic rehabilitation [1]. The hand is a vital organ that supports us in carrying out daily tasks, and fingers are a crucial component. Without fingers, hands are useless, just like a mouth without teeth. It offers us an attractive appearance and assists us in carrying out several everyday tasks. Congenital, acquired, or surgical amputation are all possible causes of loss of the finger or a portion of the digit. An individual experiences psychological distress when they must appear in public after losing a finger. The psychological anguish brought on by the loss of a finger is recovered by an aesthetic prosthesis [2].

The success of finger prosthesis depends on various factors such as diagnosis and treatment planning, material and techniques used in clinical steps and fabrication of prosthesis, and knowledge and skills of the operator. Retention of the prosthesis remains a challenging task in treating such amputated finger cases. The use of osseointegrated implants and medical-grade adhesives helps to enhance the retention of such prostheses [3]. In this case report a copper ring was used to provide the retention to the prosthesis. An extension from the copper ring was given into the tissue surface of the finger prosthesis for support which was made hollow and added to retention of the prosthesis.

## Case presentation

**Figure 1:**
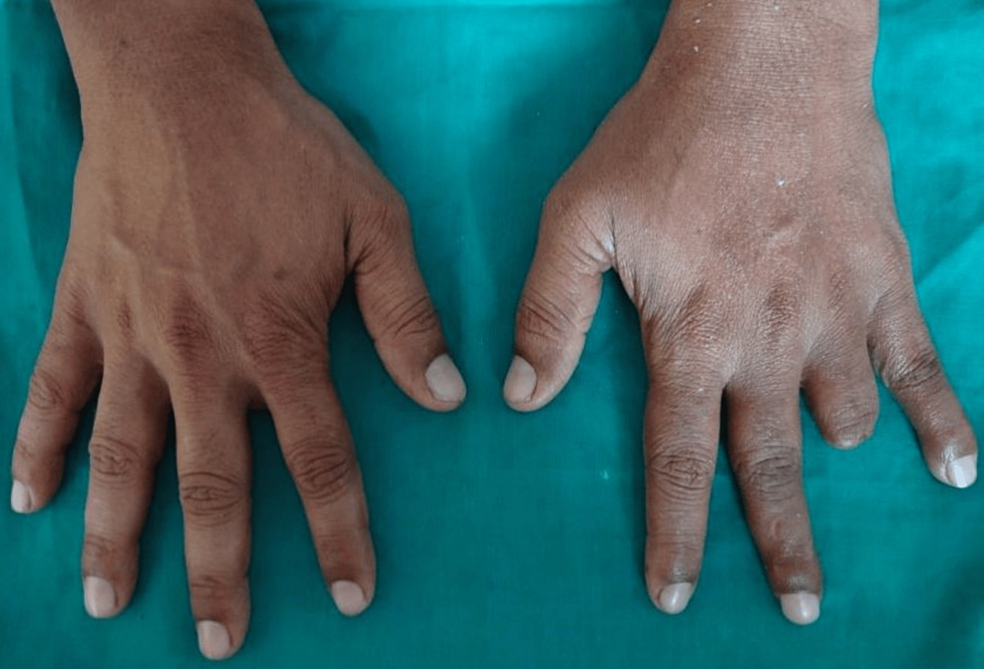
Dorsal aspect of both hands pre-operative

A 40-year-old male patient was referred to the prosthodontics department with the primary complaint of partial loss of his left ring finger. The clinical finding revealed that the intersection of the second and third phalanges was partially amputated. The amputated finger showed complete healing and the skin around it had inflammation or signs of infection and irritation (Figure 1).

**Figure 2:**
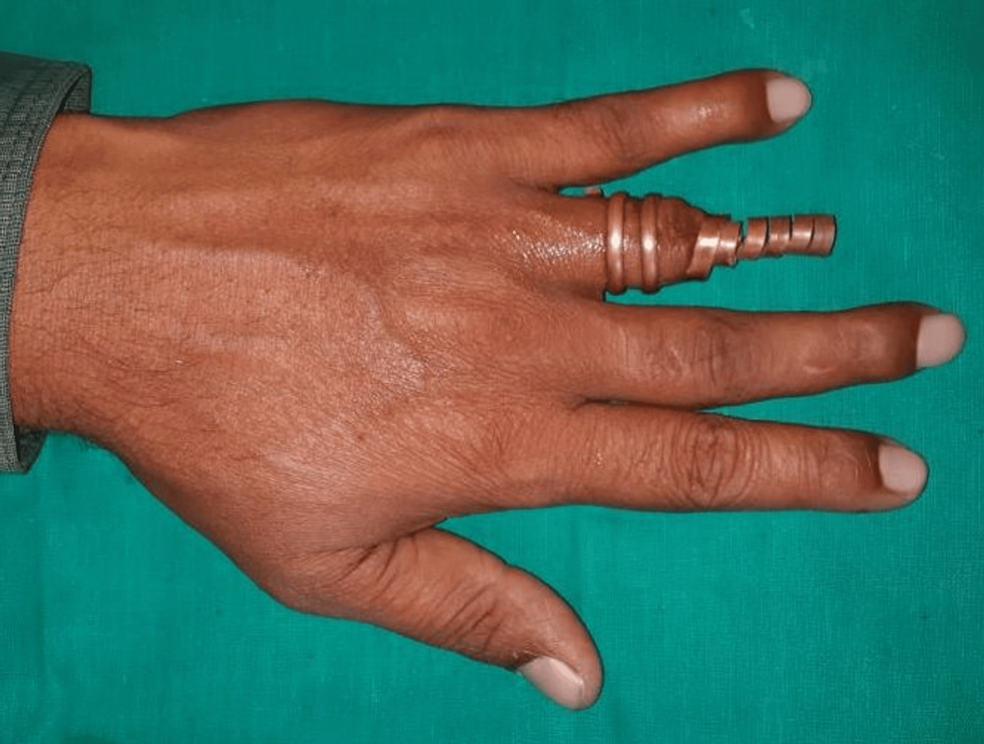
Copper ring with extension

The patient has never worn a prosthesis before. The fabrication of a copper ring with an extension into the silicon finger prosthesis was planned after a thorough diagnosis. The prosthesis' limitations were explained to the patient. A cardboard was cut in a cylindrical form with a height of 20 cm and width of 10 cm radius to record the hand to the level of the wrist. The impression of the hand was made with irreversible hydrocolloid impression material (Zelgan; DPI, Mumbai, India) to record the amputated finger. The patient was instructed to immerse the hand in the cardboard filled with alginate impression material keeping fingers in a normal resting position. The patient was asked to remove his hand slowly without tearing the material on the setting of alginate. The space obtained was filled with the type II dental stone (Kalabhai Dental, Mumbai, India). The cardboard was kept on the vibrator to avoid voids. After the complete setting of the dental stone, the mold was retrieved. In a similar manner, the impression was made for the contralateral ring finger and mold was retrieved. The mold of the contralateral ring finger helped to contour the wax pattern for the amputated finger. A copper ring was fabricated for the amputated ring finger of the patient. It was tried for the fit and then an extension was shouldered to it to extend into the prosthesis (Figure 2).

**Figure 3:**
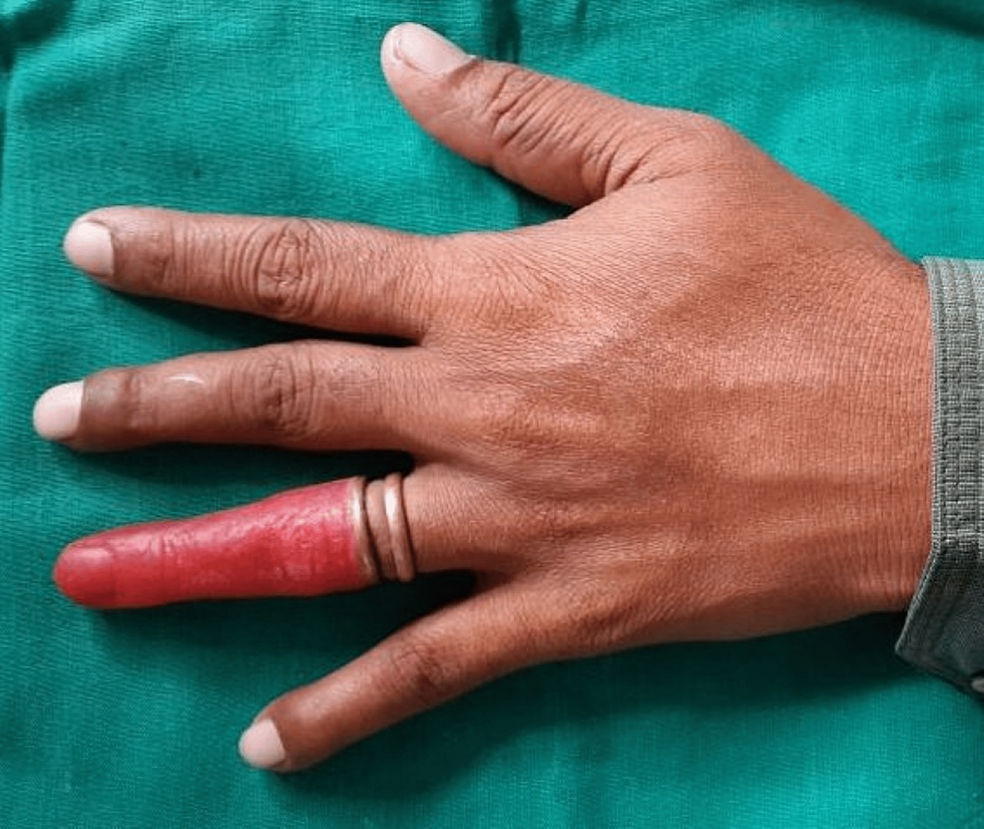
Wax pattern with a copper framework

**Figure 4:**
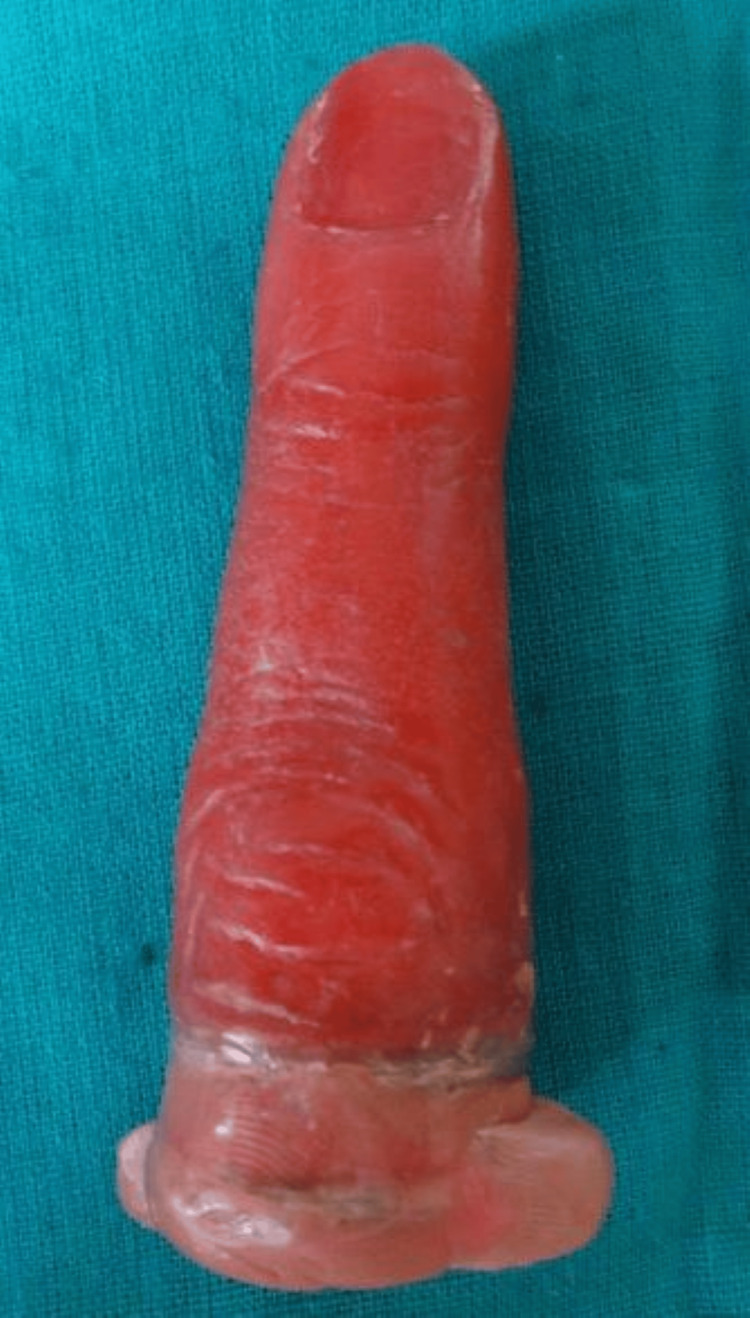
Blockout of the wax pattern with putty

The minor changes in the wax pattern were done to make it a left-sided ring finger by carving and were fitted onto the amputated finger. The wax pattern was extended into the coils of the ring and was interlocked into it. A trial was done and patient acceptance was recorded for fit, length, and width of the finger. Putty elastomeric impression material (Zhermack, Badia Polesine, Italy) was mixed and filled at the base of the finger prosthesis into the ring to prevent the dental plaster from entering the prosthesis during laboratory steps in fabricating the prosthesis (Figures 3-4).

**Figure 5:**
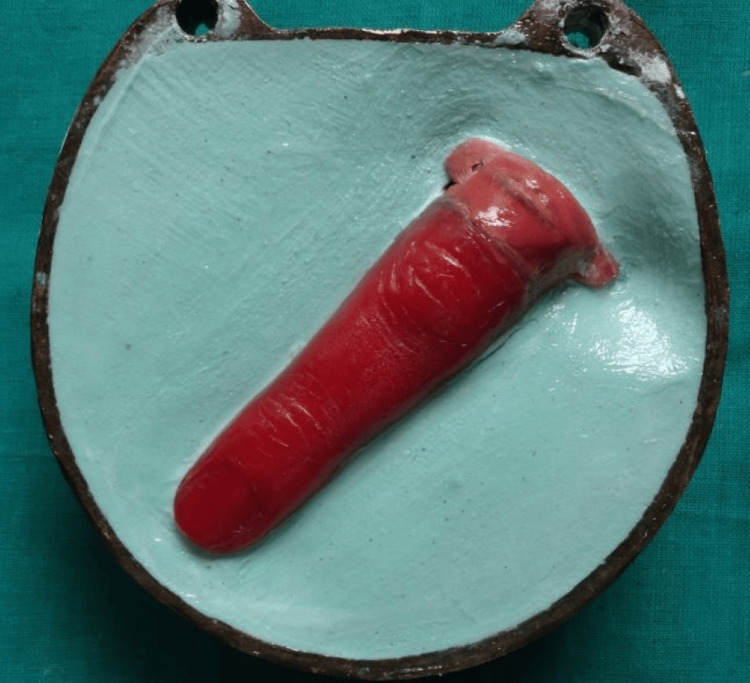
Flasking of wax pattern

**Figure 6:**
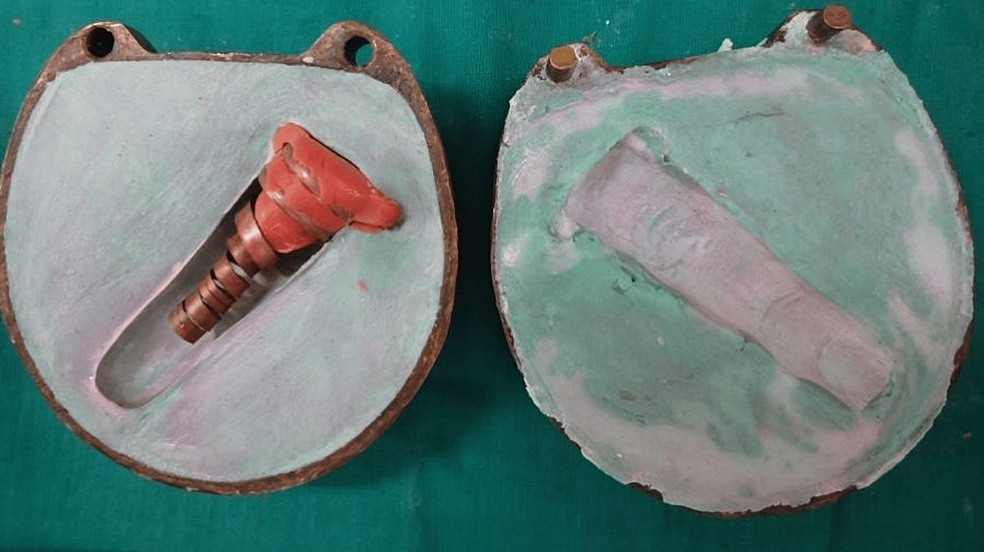
Mold obtained after dewaxing

This also helped to make the prosthesis partially hollow by restricting the material from flowing into the area with copper extension. The wax pattern along with the copper framework was invested in dental plaster to the flask. Then the conventional procedures of flasking and dewaxing were carried out (Figures 5-6).

**Figure 7:**
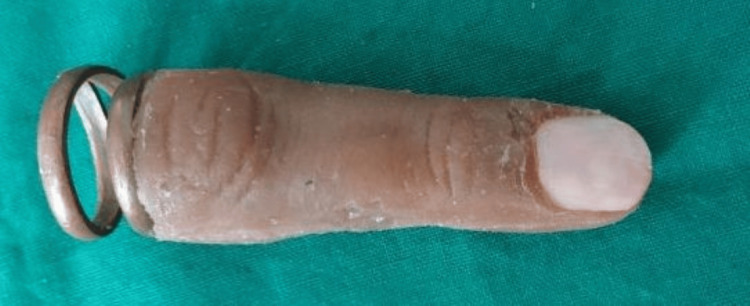
Fabricated prosthesis

**Figure 8:**
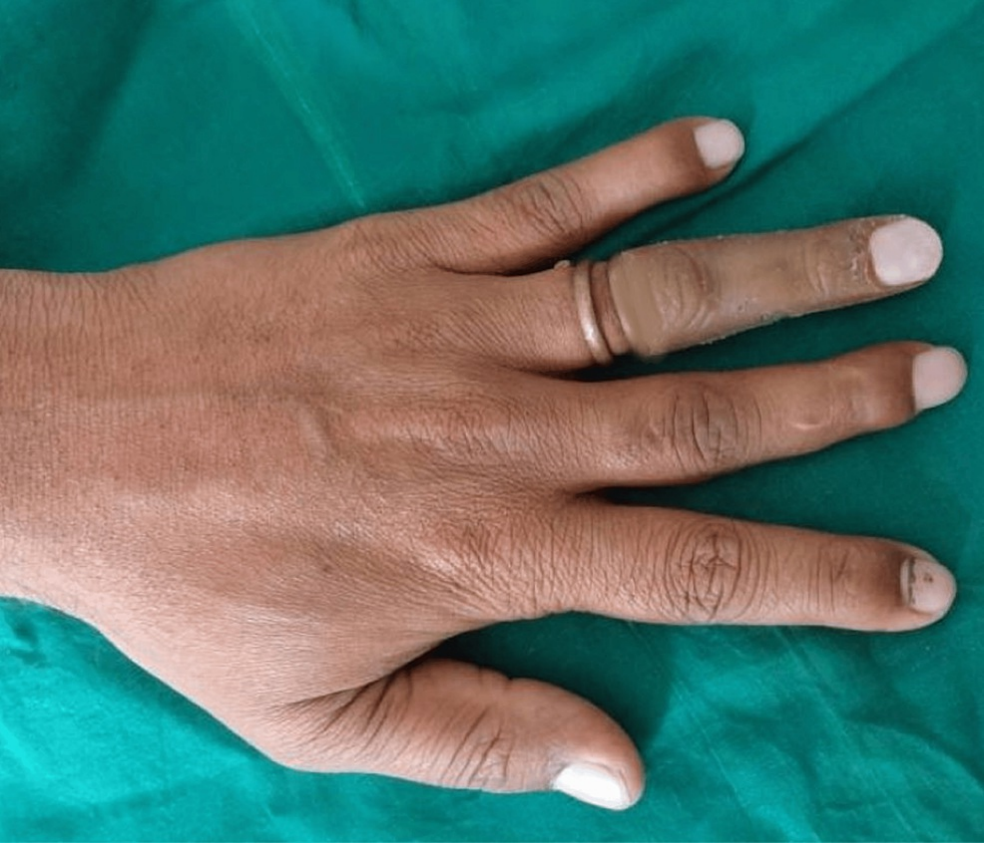
Final prosthesis – dorsal aspect

**Figure 9:**
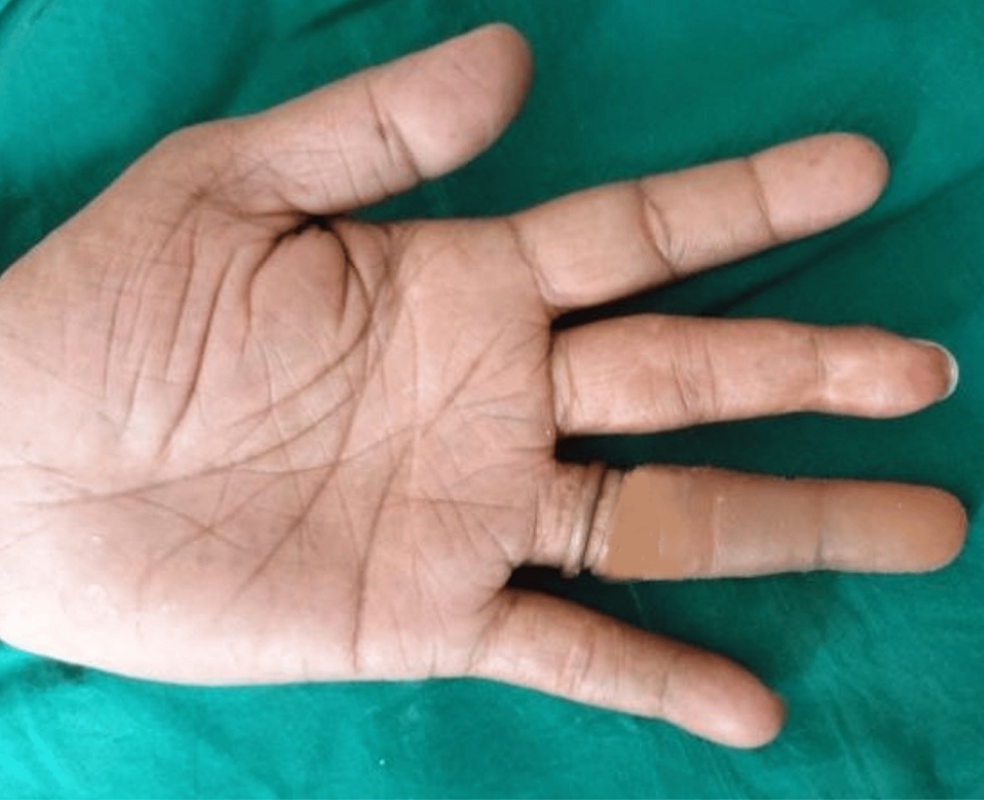
Final prosthesis – palmer aspect

Room temperature vulcanizing medical grade silicon (Technovent Ltd., Bridgend, UK) was used for packing the prosthesis. Shade matching was done separately for both the dorsal and ventral surfaces of the finger. The basic skin tone of the patient was identified and trial packing was done. On a digital scale, 20 g of catalyst and about 200 g of base were weighed out. Mixing was done with a plastic spatula on a clear clean transparent glass lab for two minutes to achieve a homogenous mix. Then, to match the skin tone, intrinsic pigment (Cosmesil Pigments; Technovent Ltd.) was weighed and added to the basic mix. Under 30 inch Hg, the silicone was vacuum mixed for 20 minutes. After that, the silicone mix was poured into the flask mold. The flask was closed and was clamped under pressure up to 30 psi. The materials were allowed to polymerize in the molds for 24 hours at room temperature (21-28°C). After the prosthesis was retrieved the putty used for blockout was removed external staining was done, and the artificial nail was attached to the finger prosthesis with cyanoacrylate (Figures 7-9).

## Discussion

In terms of both function and aesthetics, fingers are significant. Congenital conditions, injuries, infections, and tumors can all lead to finger amputations. Patients experience practical deficits and esthetic issues when one or more fingers are missing [4]. Despite the fact that microvascular surgery has saved patients' lives, prosthetic restoration remains a significant therapeutic choice for finger amputation due to technical challenges, cost constraints, and the impossibility of reconstructive surgery [5]. With improvements in microsurgical methods, certain patients could not be able to successfully repair their amputated fingers and could profit more from inactive prostheses. Concealing the use of a prosthesis has been demonstrated to be a successful way to cope because the main goal of a prosthesis is to enable the person wearing it to go unrecognized.

A finger reconstruction can be either done surgically or prosthetically. Prosthetic rehabilitation includes traditional methods of finger fabrication using heat cure acrylic resin, silicone finger prostheses, computer-aided designs of rapid prototyping and 3D printed finger prosthesis, osseointegrated dental implant supported finger prosthesis, myoelectric finger prosthesis, robotic prosthesis, and bionic fingers. As the patient reported, late surgical replantation was not possible and the patient was not willing for any invasive surgical procedure. Owing to cost and patients' desire the conventional method using the ring with silicon was opted [6].

An aesthetic and acceptable prosthesis alleviates the suffering experienced by such vulnerable people and contributes to improving their quality of life. All the steps in fabricating these prostheses are crucial. To match the shade of the prosthesis with the tone of the skin that is within the acceptable threshold is the most difficult task [7]. It is challenging to capture and reproduce the precise human skin tone in the prosthetic. Retention of the prosthesis is another problem in such cases. The use of adhesive or implants taxes the patient. The operator's skills and knowledge in handling such cases benefit the sufferer [8-10]. The objective of the present report is to provide a more straightforward method of retaining silicon finger prostheses using a copper ring framework. The copper ring framework provided retention to the prosthesis and its extension into the intaglio surface provided support to the prosthesis. The extension area of the copper ring was made hollow which reduced the bulk of the prosthesis and made the prosthesis more retentive. This custom-made silicon finger prosthesis provided esthetics and enhanced functionality by restoring the form of the finger. The patient was satisfied with the prosthesis and showed a desire to use the prosthesis.

## Conclusions

Esthetics and retention are the deciding criteria for an acceptable prosthesis. Total or partial finger amputation may have a tremendous emotional and physical impact on the individual who suffers. An effective and efficient technique using a ring to retain the finger prosthesis along with a technique of reducing the weight of the prosthesis at the same time providing extra support has been attempted and found to be successful.
